# Decentralising DR-TB care: the trade-off between quality of care and service coverage in the early phase of implementation

**DOI:** 10.5588/pha.25.0004

**Published:** 2025-09-03

**Authors:** W. Jassat, M. Moshabela, M.P. Nicol, L. Dickson, H. Cox, K. Mlisana, J. Black, M. Loveday, A.D. Grant, K. Kielmann, H. Schneider

**Affiliations:** ^1^ TB Control and Management Cluster, National Department of Health, Pretoria, South Africa;; ^2^ Genesis Analytics, Johannesburg, South Africa;; ^3^ School of Public Health, University of Witwatersrand, Johannesburg, South Africa;; ^4^ School of Nursing and Public Health, University of KwaZulu-Natal, Durban, South Africa;; ^5^ Marshall Centre, Division of Infection and Immunity, School of Biomedical Sciences, University of Western Australia, Perth, Australia;; ^6^ Division of Medical Microbiology, University of Cape Town, Cape Town, South Africa;; ^7^ Prevention of tuberculosis and other respiratory pathogens, Burnet Institute, Melbourne, Australia;; ^8^ School of Laboratory Medicine and Medical Sciences, University of KwaZulu-Natal, Durban, South Africa;; ^9^ Division of Infectious Diseases, Department of Medicine, University of Cape Town, Cape Town, South Africa;; ^10^ HIV and other Infectious Diseases Research Unit, South African Medical Research Council, Durban, South Africa;; ^11^ Centre for the AIDS Programme of Research in South Africa (CAPRISA), University of KwaZulu-Natal, Durban, South Africa;; ^12^ TB Centre, London School of Hygiene & Tropical Medicine, London, UK;; ^13^ Africa Health Research Institute, School of Laboratory Medicine and Medical Sciences, College of Health Sciences, University of KwaZulu-Natal, Durban, South Africa;; ^14^ Institute for Global Health & Development, Queen Margaret University, Edinburgh, Scotland, UK;; ^15^ School of Public Health and SAMRC Health Services to Systems Research Unit, University of the Western Cape, Cape Town, South Africa.

**Keywords:** tuberculosis, South Africa, KwaZulu-Natal, Western Cape, health system capacity

## Abstract

**BACKGROUND:**

A policy of decentralised care for drug-resistant TB (DR-TB) was introduced in South Africa in 2011. We describe a trade-off between increasing coverage of services and poor quality of care, in the early phase of policy implementation.

**METHODS:**

This was a mixed methods case study, comparing implementation in KwaZulu-Natal and Western Cape provinces; with interviews and quantitative analysis of routine DR-TB programme data. We analysed qualitative data, thematically organizing findings into inputs, processes, and outputs to explore how decentralisation influenced quality of DR-TB care.

**RESULTS:**

Decentralisation of DR-TB care expanded access across provinces but there was wide variation in pace, planning and structural readiness. Where rapid scale-up outpaced capacity-building, weaknesses in resourcing, workforce, and clinical governance compromised quality of care. Two illustrative examples highlight that decentralisation to inadequately resourced sites resulted in morbidity to patients who did not receive effective monitoring for adverse events; and decentralising services to inadequately capacitated clinicians resulted in incorrect initiation in more complex cases and late referral of clinical complications.

**CONCLUSIONS:**

Attempts to decentralise DR-TB treatment in the context of complex treatment algorithms and limited health system capacity resulted in trade-offs of care quality. We argue that quality of care should be an essential consideration in early implementation of health programmes.

South Africa is one of 30 high burden countries that account for the majority of incident drug-resistant TB (DR-TB) cases globally.^1^ Prior to 2011, provincial DR-TB centres of excellence (CoE) hospitalised patients for six months of the ‘intensive phase’ and monitored them during monthly outpatient visits for 18 months or longer during the ‘continuation phase’.^2^ Centralised DR-TB care resulted in long waiting lists and bed shortages, and patients faced significant barriers to accessing care.^3^ After discharge, the challenges of ensuring adverse events monitoring and treatment adherence support for people living far from the CoE resulted in challenges with continuity of care and poor treatment outcomes.^4^ The centralised DR-TB programme was also costly and diverted resources from national TB prevention and care.^5^ Decentralised treatment was recognised as a strategy that could overcome these challenges, and pilot projects demonstrated that decentralisation was feasible and improved access to care.^6,7^ A systematic review concluded that decentralised care was more effective than hospitalised models of care, providing improved access to diagnostic and treatment services, resulting in shorter time to treatment initiation, lower loss to follow-up (LTFU), improved treatment success, and enhanced support to people with DR-TB (PWDR-TB) and their families.^8^ Studies also modelled the cost saving of decentralised DR-TB care due to reduced hospitalisation.^5,9^

Informed by the emerging evidence, a policy of decentralised care for DR-TB was introduced in South Africa in 2011. The policy proposed transferring responsibility for the treatment of PWDR-TB to lower levels of the health care system and reducing the length of hospitalisation for those who required admission.^10^ Provinces were initially required to establish one DR-TB unit in each district. The updated policy of 2019 extended this to at least one initiation site per sub-district.^11^ However in the years following the launch of the policy, coverage of initiation sites was variable, and treatment outcomes remained poor with high LTFU and early mortality.^12,13^ TB is particularly sensitive to the quality of health systems, as people with TB must navigate a long and complex process of care-seeking, diagnosis, linkage to care, treatment initiation, and follow-up.^14^ Quality of care, defined by WHO as ‘care that is safe, effective, people-centred, timely, efficient, equitable and integrated’,^15^ is increasingly regarded as a missing ingredient in TB care.^16^ The Lancet Global Health Commission on high quality health systems estimated that 50% of TB deaths result from poor-quality care.^17^ In many settings, a ‘quality crisis’ exists, with DR-TB care remaining unsafe, inequitable, not patient-centred, and ineffective,^18^ resulting in ‘a completely broken cascade of care’.^19^

The larger study in which this analysis is embedded investigated the implementation of the DR-TB decentralisation policy in two South African provinces, Western Cape (WC) and KwaZulu-Natal (KZN).^20^ Here we describe the variable processes of decentralising DR-TB care from specialised CoEs to decentralised units, and explore the trade-offs made in balancing quality and effectiveness with increased coverage of services in the early phase of implementation. We argue that this trade-off is insufficiently acknowledged and anticipated as a problem in TB programme expansion in South Africa and globally and, consequently, is inadequately managed.

## METHODS

A mixed methods case study approach was employed, combining quantitative analysis of routine TB programme data and qualitative case studies, based on interview data, of district and provincial implementation. The case study method is well-suited to complex systems allowing for an in-depth investigation of the phenomenon in its real-life context, and the interaction of multiple factors simultaneously.^21^ The cases were defined as implementation of DR-TB decentralisation policy in the two provinces, with six embedded district cases in the WC and nine in KZN. The two provinces account for around half of the DR-TB cases in South Africa.^11^ They also provided early lessons from the implementation of pilot programmes and have a long history of decentralised DR-TB services.

Three distinct phases of DR-TB care decentralisation were observed: a first phase of piloting decentralised and ambulatory care before the policy was launched from 2008–2011, a second phase of early implementation to district sites from 2012–2015, and a third phase of wider scale-up to satellite sites from 2016 onwards.

For the quantitative analysis, we descriptively analysed routine DR-TB programme data from the electronic DR-TB information system (EDRWeb) stratified by province for the period 2008 to 2019, to evaluate changes in key indicators over time, including the number of treatment sites, number of people initiated on DR-TB treatment, and treatment success rate.

For the qualitative component, sampling was purposive, ensuring the inclusion of diverse perspectives on the DR-TB programme. In total, 84 key informant interviews were conducted with TB programme actors: six national policymakers, five provincial programme managers, 24 district and sub-district programme coordinators, 21 nurses (6 at CoE and decentralised hospitals and 15 at satellite sites), 17 doctors (5 at CoEs, 6 at decentralised hospitals and 12 at satellite sites), and 11 managers (4 at CoE and decentralised hospitals and 7 at satellite sites); and 10 in-depth interviews with TB researchers or technical experts. Interviews were conducted using an interview guide centred around a core set of themes that allowed for exploration of informants’ insights into policy implementation. Written informed consent was obtained from all participants. All interviews were audio recorded and then transcribed verbatim. Identifiers for individuals and health facilities were anonymised.

To analyse the qualitative data on the quality of decentralised DR-TB care, we applied an adapted version of the WHO framework for quality of care in health services.^22^ This structure allowed us to trace how variations in planning, infrastructure, and resources (inputs) shaped the delivery of care through staff training and clinical oversight (processes), ultimately influencing treatment success rates (outcomes). Thematic coding of interview transcripts (using Atlas Ti 4.2) was guided by these domains, enabling a structured analysis of how decentralisation impacted care quality across different phases and contexts. Coded interviews were used to derive descriptions of decentralisation and examples of care quality gaps within particular case study district and provincial contexts.

The study received ethics approval from the Biomedical Research Ethics Committee of the University of the Western Cape (BM17/7/4).

## RESULTS

The results are organised into three categories (inputs, processes, and outcomes) to illustrate how system readiness, service delivery, and clinical outcomes were interlinked. We include two illustrative examples that highlight the challenges faced during the early phase of decentralisation, related to inadequate resourcing and poorly capacitated staff.

### 1.1 Inputs: structural readiness

Provinces were expected to establish a decentralised treatment site in each district, and later in each sub-district. Over the three phases, the number of DR-TB treatment sites increased in South Africa from 17 in 2008 before the policy, to 317 in 2015 following early implementation, to 420 by 2019 in the wider scale-up. This scale up occurred with considerable variation in the nature, degree and speed of decentralisation between and within provinces. In the WC, a decentralised treatment programme in primary health care (PHC) clinics was first implemented in 2008 in urban Khayelitsha, then rapidly scaled up across the urban City of Cape Town. Rural districts maintained hospital-based models with down-referral and outreach support to clinics until 2016, when initiation of treatment in clinics began. By 2019, 248 health facilities in the WC were initiating DR-TB treatment, representing 51% of 489 public health facilities (Figure panel A). These were predominantly clinics rather than hospitals, and in rural areas, supported through an outreach model.

**FIGURE.**
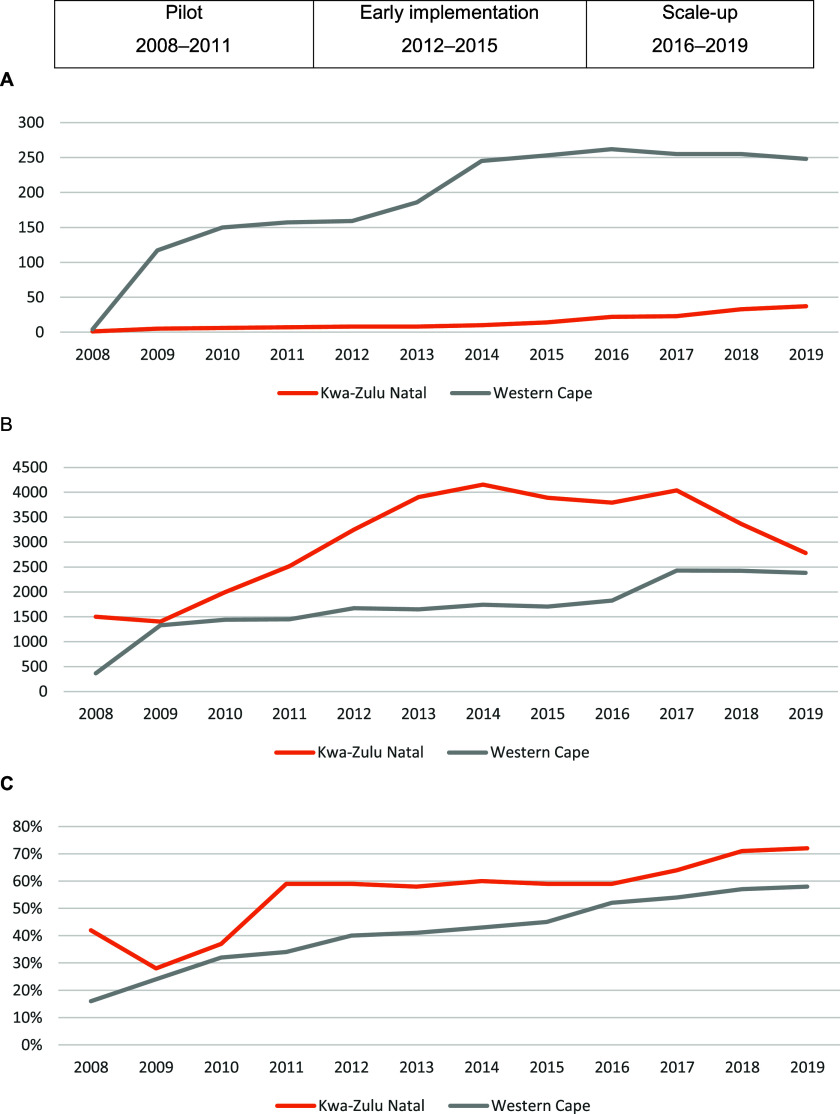
Performance of the DR-TB programme during pre-policy (2008–2011), early implementation (2012–2015), and scale-up (2016–2019) phases, in Western Cape and KwaZulu-Natal, depicting (**A**) number of DR-TB initiating sites established, (**B**) number of people initiated on DR-TB treatment, and (**C**) DR-TB treatment success rate (source EDRWeb).

Ambulatory treatment was first introduced in KZN in uMzinyathi district as a pilot programme in response to an XDR-TB outbreak in 2005. This was followed by the establishment of four decentralised DR-TB sites in district hospitals between 2008 and 2011. Once each district had a DR-TB site, they began down-referral to satellite sub-district sites (usually hospitals), then capacitated them to become initiating sites. By 2019, KZN had 37 initiating sites, representing 1% of 3,211 public health facilities (Figure panel A).

As the DR-TB burden increased, decentralisation in both provinces was inevitable, and with time, a degree of harmonisation was achieved as most districts in both provinces moved towards implementing a fully decentralised model to satellite sites. However, as indicated, the approaches in the early phase of implementation were different and occurred with varying planning and preparation, resourcing, and health care worker capacity, which impacted quality of care.

### 1.2. Inputs: resource challenges

Clinicians expressed resistance to establishing decentralised sites in their hospitals due to the lack of equipment for monitoring, challenges with infrastructure, insufficient space in the facilities, and inadequate infection control. With the introduction of this policy, newly established decentralised sites would require equipment, drugs, and staff for diagnosis, monitoring, and treatment support. Examples include audiometry to monitor patients on ototoxic drugs for hearing loss, and electrocardiograms to monitor patients started on bedaquiline for cardiac side-effects.^8^

Availability of equipment depended on the level of care (hospital vs PHC) and phase of implementation. Audiometry and electrocardiographs would be available at district and regional hospitals but may not be available at PHC facilities. During the early phase of implementation, provinces received donor funding to procure Kuduwaves (audiometer for monitoring ototoxicity and detecting early hearing loss). Decentralised sites established later did not benefit from donated equipment. As a result, PWDR-TB were not monitored effectively for adverse events on toxic drugs (Box 1). Managers and clinicians tasked with implementing the policy felt that they were expected to implement policy but were not provided with the resources required. A KZN DR-TB doctor remarked:

The province comes with a policy on paper and they say, ‘Do 1,2,3’, but they don’t come with all of the necessary things that you need on the ground.

BOX 1. Hearing loss in a rural DR-TB unit related to lack of equipment for decentralised care.

**Background:** District A was one of the last in the province to establish a DR-TB site in 2015. In 2016, a story was posted in the local media that  100 PWDR-TB treated at the hospital had developed hearing loss.

**Observed challenges**: The hospital had inadequate infrastructure and was unable to admit PWDR-TB, and therefore could not start patients on  bedaquiline. Aside from not being able to change PWDR-TB from ototoxic regimens containing injectables, it emerged that they were not conducting hearing tests during this period, because the Kuduwave was reportedly sent for calibration, with no replacement equipment available. TB clinicians thus continued to initiate patients without baseline and follow-up hearing tests.

What happened was between 2015 and 2016, no baseline hearing tests were done before initiating patients on kanamycin because there was no Kuduwave to test patients before starting them on treatment … We did have a Kuduwave but it was gone for calibration or something like that … No plan was made (by the district). We just started initiating. (rural DR-TB unit nurse)

**Implications:** The option of referral to a nearby regional hospital, which had the equipment and an audiologist, was also not pursued.

During that period … a patient will go three or four months without any audio being done … We’ve got Hospital X that has an audiologist and  everything there, but if you look at the clinical notes, they were not being referred for audiology. (rural TB coordinator)

After the story was published in the media, the health department procured new audiometry equipment and all PWDR-TB began having baseline and  follow-up audiometry. It was clear that having a site not adequately resourced to deliver DR-TB care, resulted in significant morbidity to patients who did not receive effective care.

### 2. Processes: health workforce and clinical governance

Decentralising too quickly impacted quality by not adequately building capacity and systems to supervise clinicians at lower levels for delivering DR-TB care. New decentralised sites were reported to be inadequately staffed, had difficulty retaining trained doctors and nurses especially in rural areas, and experienced increased workloads following integration of DR-TB into mainstream PHC services. Coverage of training was uneven across districts, and with attrition, new staff were less likely to be trained. Once-off training did not adequately prepare clinicians, who required ongoing systems of mentoring, oversight, and skilled support. As a result, nurses and doctors at satellite sites often did not know or follow the treatment guidelines, and several PWDR-TB were not placed on correct regimens, monitored for side-effects, or referred early for management of complications (Box 2). Many informants believed that over-hasty decentralisation from CoEs to poorly capacitated clinicians without the necessary support and outreach, would impact quality of care. Some TB programme managers resisted pressures to increase the pace of decentralisation.

We have been nailed … by the province and national, to say the pace at which we are decentralising is not what they expected. We’ve been saying that we will never ever decentralise at the expense of quality. (KZN rural district TB coordinator)

BOX 2. Clinical management gaps related to inadequately trained and supported health care workers.

**Background:** Rural district B established a DR-hospital in 2011 that served as a referral service for DR-TB patients across the district. In 2012, an  enthusiastic medical manager from the DR-TB hospital capacitated satellite sites to begin initiating PWDR-TB predominantly through a nurse-led model. Several PHC clinics were rapidly supported to become satellite sites. The medical manager had been mentoring nurses and doctors, but when the manager resigned and took a position in another district, that mentoring and support ended. Trained doctors resigned and new doctors were less interested in DR-TB, and as a result, patients were mainly being managed by nurses who no longer received any mentoring.

**Observed challenges:** An example of the impact on quality of care was of patients with renal dysfunction being initiated by nurses at the satellite site  despite the guidelines limiting nurses’ scope to initiating uncomplicated patients. Patients deteriorated clinically and were referred late to the district DR-TB site.

Patients are developing complications out there. They are not sent here to the doctor, to manage them... When the doctor looks at their results, the doctor can see this patient was initiated on treatment yet the U&E [kidney function test] of this patient did not allow it … These people are trained … You know that if the patient is having renal failure, you should not initiate these drugs. (rural DR-TB unit manager)

Unsupervised nurses at satellite sites did not adequately monitor patients, and patients with complications were identified and referred late.

If a patient is having hearing loss, you can’t just develop that overnight, there are some signs. If the nurse is injecting this patient daily, a patient cannot come here with total hearing loss in both ears. (rural DR-TB unit manager)

**Implications:** When the new DR-TB medical manager started visiting satellite clinics, they discovered that nurses had been trained, but treatment  guidelines were not being followed. Nurses relied on telephonic consults with CoE doctors. Most TB clinicians and managers believed this was inadequate and that there should, in fact, be a doctor on-site at satellite units.

If this satellite programme was working well according to my view, there was going to be a doctor in that satellite who is attached to that clinic, who was assisting the nurses, because we as nurses we know how far we can go, and the doctor must come in there. (rural DR-TB unit nursing manager)

There were clear benefits of decentralising DR-TB care, for managing the patient load and bringing care closer to patients, but without proper  mentoring, local clinicians and managers believed patients would have been better off waiting for care at the district DR-TB unit rather than being treated at poorly capacitated and inadequately supported satellite sites.

Clinical governance interventions, including mentoring doctors and nurses at satellite sites, conducting quality of care audits, and establishing forums for coordinating clinical care, were implemented variably across the two provinces. TB experts and managers affirmed the importance of clinical governance, and many made a purposeful effort to ensure decentralisation occurred slowly, while building capacity, empowering staff, and ensuring good quality of care.

You’ve got one good chance to cure the patients. So it is decentralised there, but I’m not sure that the clinical governance and the quality is there … that is why in this area I firmly stand my ground to say we are going to do it slowly, over time, over years, we are going to build capacity so that the doctors don’t feel like we have dumped something very complex on them. (WC rural DR-TB unit manager)

### 3. Outcomes

Despite both provinces scaling up the number of treatment sites, and the numbers of people initiated on DR-TB treatment increasing steadily in the pilot and early implementation phases, quality was compromised in the early phases due to weak inputs and inadequate support systems. KZN, which adopted a slower, more structured rollout, achieved better treatment outcomes than WC, where rapid expansion may have outpaced capacity-building (Figure panel A and C). The findings underscore that expanding access without building a foundation for quality – including workforce, infrastructure, and governance – risks undermining patient outcomes.

## DISCUSSION

This study examined the decentralisation of DR-TB care in two high-burden South African provinces, using a health systems framework that categorised findings into inputs (structural readiness), processes (clinical governance), and outcomes. While both provinces achieved considerable scale-up in the number of treatment sites, our findings reveal that uneven structural investments, inconsistent health workforce development, and variable clinical governance resulted in trade-offs of care quality. These trade-offs were more likely during the early phase of implementation when there was pressure for rapid scale up of a complex intervention. The DR-TB decentralisation policy prioritised expanding coverage by establishing decentralised sites in each district to improve access to care, reduce patient barriers such as cost and transport, and provide patient-centred care closer to home. However, political pressure to meet decentralisation targets compelled provinces and districts to report on the number of new sites established, rather than on the quality of services provided. As a result, implementation proceeded rapidly, often without adequate planning, and before the necessary training and resources outlined in the policy were in place. This rollout took place within an already overburdened health system facing chronic resource constraints and systemic weaknesses. Consequently, the quality of care was compromised in some settings, by poorly capacitated and unsupported health care workers operating within strained health service infrastructure. Although efforts were made to uphold care quality, these were either inadequate or not sustained.

Other studies have noted the threats to quality when moving care from specialised DR-TB hospitals to district hospitals in South Africa. In decentralised sites new to implementing DR-TB treatment, clinical management errors, inadequate monitoring of adverse events, and suboptimal treatment occurred in half of the cases reviewed.^23^ In a study evaluating implementation in 22 decentralised sites in four provinces, HCWs reported that they had no prior experience with PWDR-TB and were offered little support, and they faced difficulty accessing radiology, audiology, and transport which hampered quality of care.^24^ In contrast, DR-TB treatment models that embrace continuous quality improvement approaches have been associated with favourable treatment outcomes.^25^ Studies from other low and middle-income country settings demonstrated that initial and ongoing training, mentoring, and supervision of HCWs were essential to providing quality DR-TB care. In Uganda, decentralisation to community-based services resulted in improved outcomes because they focused not only on establishing decentralised services, but on quality-related strategies, including peer-to-peer mentorships and coaching, standard operating procedures, access to treatment monitoring investigations, management of TB drugs, strengthened data management, and cohort reviews.^26^ In Tanzania, a national DR-TB continuous learning approach involving a standardised training package, on-the-job mentoring, and follow-up supervision, resulted in good treatment outcomes despite health system challenges.^27^ In Ethiopia, improved treatment success was achieved through a comprehensive programme strengthening approach, including monthly mortality audits and continuing medical education sessions.^28^

The tension between ‘efficiency’ (access and equity) and ‘quality of care’ (safety and patient-centredness) is a well-recognized challenge in health systems. The current approach to global TB control focused on expanding coverage of TB services rather than on the quality of TB care routinely offered to patients, needs to be reconsidered.^29^ National TB programmes will therefore need to think beyond access and coverage of healthcare services, and begin to systematically measure and improve quality of TB care.^29^ A case study in Pakistan concluded that initial gains from DR-TB decentralisation were not sustained because the programme prioritised ‘materialities’ (materials, infrastructure, and technologies) and not competencies (knowledge, skills, and processes) required for high-quality care.^30^ Although quality and performance indicators may be difficult to measure, they are essential to capturing whether DR-TB services are effective. This aligns with the concept of ‘effective coverage’ introduced by the WHO, which combines need, utilisation, and quality to assess health system performance. The Lancet Commission on TB similarly argues that governing for quality demands a shift from monitoring treatment coverage to measuring the effective coverage of TB care.^19^ High coverage of poor-quality care is not an acceptable outcome and is in fact ‘ineffective, wasteful, and unethical’.^14^

## CONCLUSION

Successful decentralisation of complex programmes like DR-TB care depends not only on expanding access, but also on preserving the programme characteristics—such as strong clinical governance—that are essential for achieving good outcomes, especially when care is delivered by less specialised staff at peripheral sites. The inherent trade-off between service coverage and quality of care must be explicitly recognised and systematically assessed to enable effective implementation. We advocate for a shift in perspective: from focusing solely on coverage to prioritising effective coverage, which integrates access, utilisation, and quality, as the true measure of success in health programmes.
